# Two new endemic genera and a new species of toad (Anura: Bufonidae) from the Western Ghats of India

**DOI:** 10.1186/1756-0500-2-241

**Published:** 2009-12-07

**Authors:** SD Biju, Ines Van Bocxlaer, Varad B Giri, Simon P Loader, Franky Bossuyt

**Affiliations:** 1 Systematics Lab, Centre for Environmental Management of Degraded Ecosystems (CEMDE), School of Environmental Studies, University of Delhi 110 007, India; 2 Biology Department, Unit of Ecology & Systematics: Amphibian Evolution Lab, Vrije Universiteit Brussel (VUB), Pleinlaan 2, B-1050 Brussels, Belgium; 3 Collections Department, Bombay Natural History Society (BNHS), S.B. Singh Road, Mumbai 400 001, India; 4 Institute of Biogeography, Department of Environmental Sciences, University of Basel, Klingelbergstrasse 27, 4056 Basel, Switzerland

## Abstract

**Background:**

Bufonidae are a large family of toads with a subcosmopolitan distribution. Recent molecular phylogenetic analyses have revealed a radiation of toads (Adenominae) with distinct adult and larval ecomorphs on the Southern parts of the Indian subcontinent. The Indian torrential species "Ansonia" ornata has a basal position in this clade and does not group with South-East Asian Ansonia. Additionally, the nested position of "*Bufo*" *koynayensis *and an undescribed sister species, and their distinct ecologies including a non-typical egg-laying strategy within bufonids, support the recognition of a second distinct genus. In this paper we describe two new genera and one new species from the Adenominae clade.

**Findings:**

Ansonia ornata Günther, 1876 "1875" is transferred to Ghatophryne gen. nov., a genus of torrentially adapted toads that are endemic to the Western Ghats of India. On the basis of close morphological resemblance and distribution, Ansonia rubigina Pillai and Pattabiraman, 1981 is provisionally transferred to this new genus. The Western Ghats endemic toad Bufo koynayensis Soman, 1963 is transferred to a new genus Xanthophryne gen. nov. Based on molecular and morphological evidence, we additionally describe a new species, Xanthophryne tigerinus sp. nov., from Amboli in the Western Ghats.

**Conclusion:**

The descriptions and subsequent taxonomic changes we propose result in three genera of bufonids recognised as being endemic to the Western Ghats (Ghatophryne gen. nov., Xanthophryne gen. nov. and Pedostibes), and one to Sri Lanka (Adenomus). The spatial distribution, and arrangement of these lineages at the base of Adenominae diversification, reflects their Early Neogene isolation in the Western Ghats-Sri Lanka hotspot.

## Introduction

Bufonidae are a family of toads with over 500 extant species distributed on most continents. Current opinions on bufonid taxonomy are very divergent, and range from the recognition of multiple genera [1], to favouring a subcosmopolitan genus Bufo with plenty of subgenera [2]. Although the phylogenetic relationships of bufonids have been studied intensively [1,3,4], the evolutionary position of species on the Indian subcontinent had remained unclear. However, recent molecular phylogenetic analyses revealed that these toads belong to a radiation containing distinct ecomorphs in adult and/or larval forms [5]. The early diversification of this clade, for which the name Adenominae is available [6], has been reconstructed as occurring on the Southern parts of the Indian subcontinent. Early diversification of Adenominae, timed at the early Miocene, led to several endemic lineages ("*Ansonia*" ornata, *Pedostibes tuberculosus*, the "*Bufo*" *koynayensis *group and *Adenomus*) [5] and to the origin of the more widespread *Duttaphrynus *clade. The unexpected evolutionary position of "Ansonia" ornata and "Bufo" koynayensis (see abstract) requires recognition of two new genera to accommodate these Western Ghats endemic species and their relatives.

## Methods

Collection of amphibian specimens was made during fieldtrips in the Western Ghats between 1997 and 2002. Specimens were preserved in 5% formaldehyde for 2 days, and subsequently transferred to 70% ethanol. Measurements were taken to the nearest 0.1 mm, using a digital slide-caliper or a binocular microscope with a micrometer ocular. The description (all measurements in mm) of the types follows terminology used elsewhere [7]. To comply with regulations of the International Code of Zoological Nomenclature we have deposited copies of this article at the following publicly accessible libraries: Bombay Natural History Society, Mumbai, India (BNHS); Koninklijk Belgisch Instituut voor Natuurwetenschappen, Brussels, Belgium (KBIN); Natural History Museum, London, UK (BMNH); American Museum of Natural History, New York, USA (AMNH); Museum National d'Histoire Naturelle, Paris, France (MNHN); Russian Academy of Sciences, Moscow, Russia (RAS).

## Descriptions

### 1. *Ghatophryne *gen. nov. (Figure 1)

**Figure 1:**
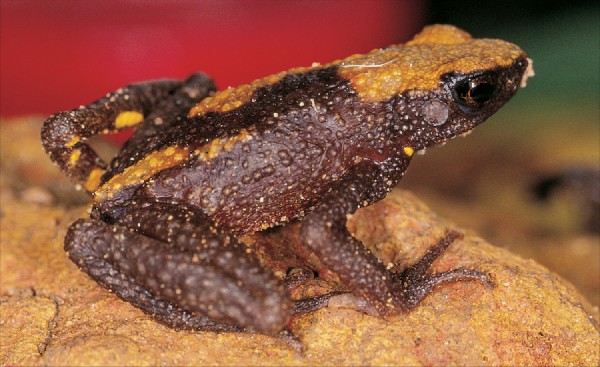
***Ghatophryne ornata *specimen SDB 6361, adult male, SVL 29.2 mm, collected from Kurichiyarmala, 11°35'N, 75°58'E, Wayanad, Kerala**.

#### Etymology

The generic epithet is derived from the words 'Ghats' and 'phryne'. The former is the Sanskrit word for 'steps' and refers to the Western Ghats mountain range, where this genus is endemic, while 'phryne' means toad in Greek.

#### Type species

*Ansonia ornata *Günther, "1876" 1875, by original designation.

The original description of this species was based on nine syntypes (BMNH 1947.2.20.65-1947.2.20.73) collected in "Bramagherries" (= Brahmagiri Hills, Coorg) by R. H. Beddome. We hereby designate one of them, BMNH 1947.2.20.66 (ex BMNH 74.4.29.945), an adult male, SVL 29.0, as lectotype of this nominal taxon.

#### Diagnosis and comparison

Ghatophryne can be distinguished from other bufonid genera by the combination of the following characters: small-sized adults (male SVL 26.9-30.1, N = 5; female 35.0, N = 1); dorsum reddish brown, ventrum dark brownish black with prominent yellowish-orange spots; head without cranial ridge, lack of parotoid glands; fingers free, toes medium webbed; skin on dorsum with sparsely granular projections especially on anterior half of the body; eggs non-pigmented, tadpoles with a suctorial disk, adapted to mountain streams throughout the life cycle (SD Biju, personal observations). Ghatophryne can be characterized in a phylogenetic framework as the most inclusive clade that contains *Ansonia ornata *Günther, "1876" 1875, but not *Bufo melanostictus *Schneider, 1799 and *Ansonia hanitschi *Inger, 1960 [5].

*Ghatophryne *is morphologically very similar to species of *Ansonia*, but is endemic to the southern part of the Western Ghats, while *Ansonia *is known only from South-East Asia (Sumatra, Borneo and Philippines).

#### Contents

Ghatophryne ornata (Günther, "1876" 1875) **comb. nov.**, Ghatophryne *rubigina *(Pillai and Pattabiraman, 1981) **comb. nov. **The latter is provisionally included based on distributional data and morphological resemblances [8].

#### Distribution

Ghatophryne has only been reported from Kerala and Karnataka in the Western Ghats of India.

#### Description of lectotype of Ansonia ornata Günther, "1876" 1875

Medium sized frog (SVL 29.0), body rather elongate. Head slightly longer than wide (HL 8.9; HW 8.5; MN 7.2; MFE 5.4; MBE 1.3); outline of snout in dorsal view nearly oval, its length (SL 4.5) larger than the horizontal diameter of the eye (EL 3.7); loreal region vertically acute; distance between anterior margins of eyes (IFE 4.6) 1.8 times in distance between posterior margins of eyes (IBE 8.4); interorbital area (IUE 2.8) broader than upper eyelid width (UEW 2.3); tympanum (TYD 2.7) rather indistinct; supratympanic fold rather indistinct; parotoid glands absent; parietal ridges absent; tongue elongate. Forearm (FLL 7.5) shorter than hand (HAL 8.8; TFL 4.0), strong; relative length of fingers, shortest to longest: I < II < IV < III, tip of fingers enlarged, rounded, without distinct grooves, without lateral dermal fringe, webbing absent; subarticular tubercles weakly developed; supernumerary tubercles weakly developed; palmar tubercle absent, nuptial pads present. Hind limbs moderately long, shank (ShL 14.1) longer than thigh (TL 13.3), longer than distance from base of inner metatarsal tubercle to tip of toe IV (FOL 12.6), shorter than distance from heel to tip of toe IV (TFOL 19.2); relative length of toes, shortest to longest: I < II < V < III < IV, tips of toes enlarged, rounded, webbing reduced, reaching up to second subarticular tubercle on both sides of toe IV; dermal fringe along toe V absent; subarticular tubercles distinct; supernumerary tubercles weakly developed; inner metatarsal tubercle elongate (IMT 1.5), outer metatarsal tubercle smaller but distinctly raised. Skin of snout, between eyes, and upper eyelids shagreened to sparsely granular, side of head, back, flanks and dorsal part of limbs sparsely granular.

*Colour of lectotype*: in preservation dorsally uniform light greyish brown with a dorsal light yellowish brown patch; ventral side light brown with white irregular spots.

#### Other specimens studied

Ovary of single female specimen (SDB 022) had 35 non-pigmented, creamy white eggs, 1.3 ± 0.4 mm in diameter. Dorsum can have continuous (SDB 6362) or discontinuous (SDB 6361) yellowish bands.

### 2. *Xanthophryne *gen. nov

#### Etymology

derived from two Greek words, 'xanthos' meaning yellow, and 'phryne' meaning toad.

#### Type species

Bufo koynayensis Soman, 1963, by original designation, ZSIC A1784, Shivaji Sagar lake at Koyna (= Humbarli Village), Maharashtra, collected by P. W. Soman.

#### Diagnosis and comparison

*Xanthophryne *can be distinguished from other bufonid genera by the combination of the following characters: small-sized adults (male SVL 26.5-32.9, N = 12; female SVL 33.3-35.3, N = 3) having light brown dorsum with a suffusion of dull chrome-yellow; head with discontinuous and weak canthal and preorbital ridges on the anterior part, flanks and sides of the abdomen have chrome-yellow patches, or sometimes 2-4 continuous bands; tympanum indistinct, rather weak parotoid glands; toes and fingers without webbing, tips rounded; eggs in clutches. *Xanthophryne *can be characterized in a phylogenetic framework as the most inclusive clade that contains Bufo koynayensis Soman, 1963 but not Bufo melanostictus Schneider, 1799 and Bufo kelaartii Günther, 1858.

#### Contents

**Figure 2:**
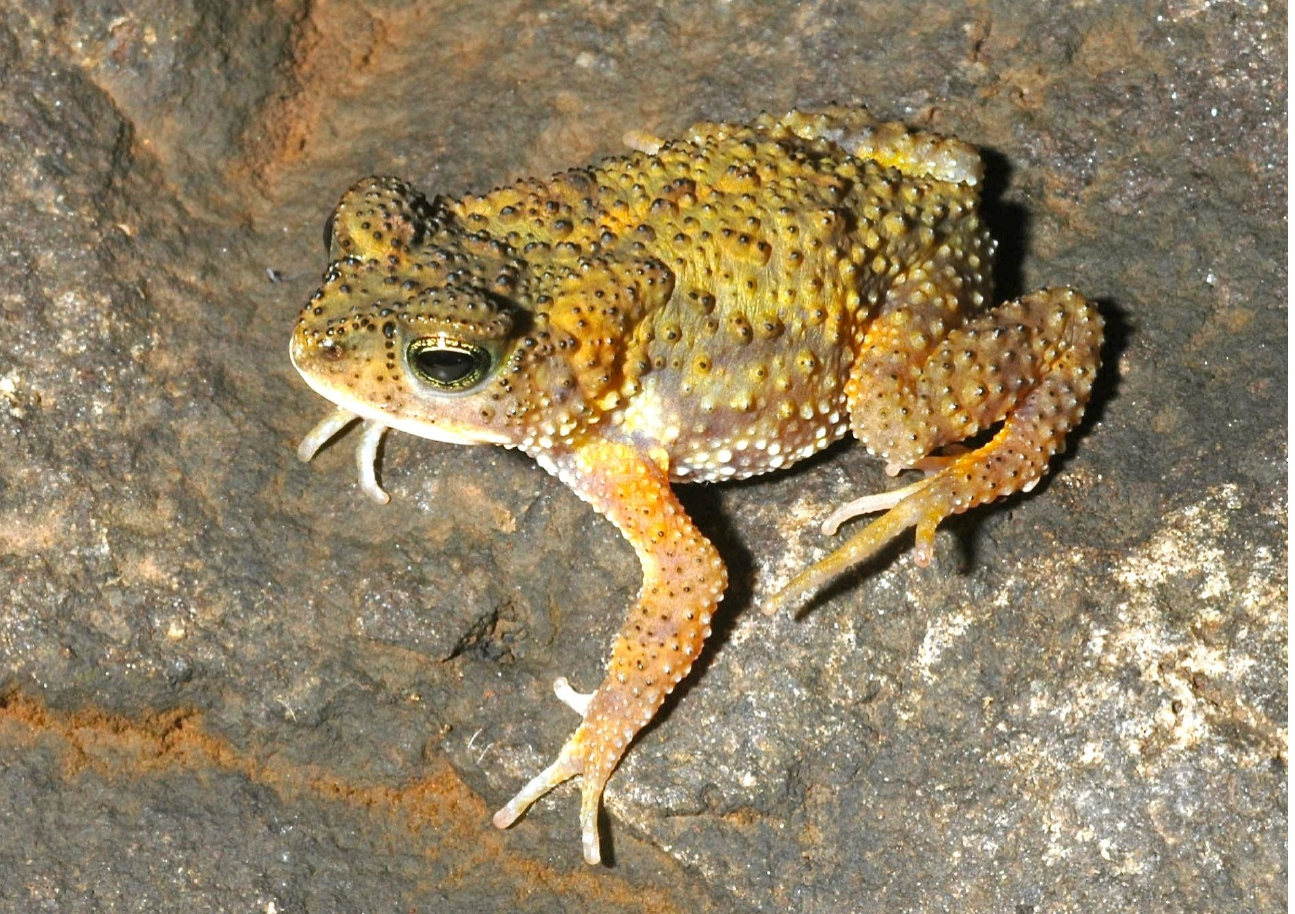
***Xanthophryne koynayensis*, a male adult (SDB 6040) from the type locality**.

**Figure 3:**
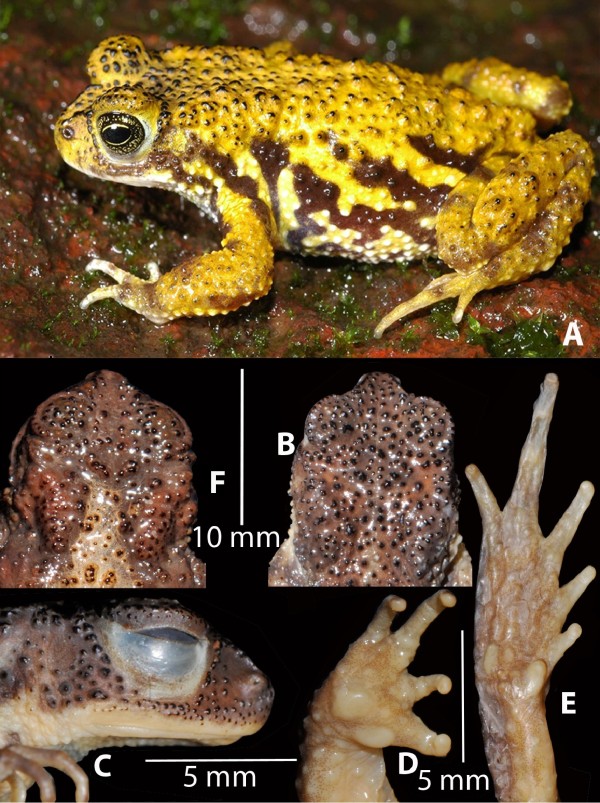
***Xanthophryne tigerinus *sp. nov (holotype, BNHS 5175)**. A, holotype in life; B, dorsal view of head showing prominent granular projections with horny spinules; C, lateral view of head; D, ventral view of hand; E, ventral view of foot; F. *Xanthophryne koynayensis*, dorsal view of the head, showing scattered granular projections with horny spinules.

*Xanthophryne koynayensis *(Soman, 1963) **comb. nov. **(Figure 2) and Xanthophryne tigerinus **sp. nov**. (Figure 3A)

#### Distribution

*Xanthophryne *has only been reported from the northern part of the Western Ghats of India.

### *3. Xanthophryne tigerinus *sp. nov.(Figure 3A-E)

#### Etymology

The species epithet *tigerinus *is attributed to this species due to similarity in the species colour pattern to the prominent lateral stripes in Tigers (*Panthera tigris*).

#### Type material

Holotype, BNHS 5175, an adult male, SVL 30.5, collected by SDB and VG on 23 August 2002 from Amboli, 15°56'N, 74°00'E, about 720 m asl, Sindhudurg, Maharashtra, India; paratypes, BNHS 5177, an adult male, BNHS 5176, an adult female, collected along with the holotype; BNHS 4064, an adult male, BNHS 4063, an adult female, collected by VG & Hegde on 9 September 2000, BNHS 4208-4209, two males, BNHS 4207, an adult female, collected by Kehimkar and Agarwal on 16 August 2003.

#### Diagnosis and comparison

*Xanthophryne tigerinus *can be distinguished by the following combination of characters: (1) medium size, male adult SVL 27.8-32.9, female adult SVL 33.3-35.3; (2) body rather elongate; (3) presence of discontinuous canthal and preorbital ridges; (4) stripes on lateral and dorsal side; (5) absence of webbing between fingers and toes.

*Xanthophryne tigerinus *sp. nov. (Figure 3A-E) differs from *X. koynayensis *(Figure 2, 3F) by the presence of a denser arrangement of granular projections with horny spinules on dorsal and lateral parts of head, back and flank; more prominent canthal and preorbital ridges; snout longer than eye length (SL 4.0 ± 0.3 mm, EL 3.4 ± 0.5 mm, *N *= 5, male) vs. snout shorter than eye length (SL 3.3 ± 0.3 mm, EL 4.3 ± 0.2 mm, *N *= 7, male); shank longer than thigh (ShL 11.5 ± 0.6 mm, TL 9.9 ± 0.4 mm, *N *= 5, males) vs. shank about equal to thigh (ShL 11.1 ± 0.7 mm, TL 11.1 ± 0.8 mm, *N *= 7, male); foot length longer than shank and thigh (FOL 12.8 ± 0.6, *N *= 5, male) vs. equal to shank and thigh (FOL 11.2 ± 0.7, *N *= 7, male).

#### Description of the holotype

Medium sized frog (SVL 30.5), body rather elongate (Figure 3A). Head length subequal (HL 10.1) to head width (HW 10.3; MN 8.8; MFE 7.2; MBE 4.2) (Figure 3B, C); outline of snout in dorsal view nearly oval, in ventral view oval to pointed, its length (SL 4.1) longer than the horizontal diameter of the eye (EL 3.1); loreal region vertically acute; distance between anterior margins of eyes (IFE 4.6) 1.9 times in distance between posterior margins of eyes (IBE 8.7); interorbital area (IUE 2.3) less than upper eyelid (UEW 3.1); tympanum rather indistinct; supratympanic fold absent; parotoid glands present, rounded; canthal and preorbital ridges well developed, continuous; tongue entire, oval. Forearm (FLL 6.1) shorter than hand (HAL 7.0; TFL 3.5); relative length of fingers, shortest to longest: I < II < IV < III (Figure 3D), tip of fingers enlarged, rounded, without distinct grooves, without lateral dermal fringe, webbing absent; subarticular tubercles weakly developed; prepollex rounded, prominent, palmar tubercle one, rounded, prominent; supernumerary tubercles weakly developed; nuptial pads present. Hindlimbs moderately long, shank (ShL 11.8) longer than thigh (TL 10.1), shorter than distance from base of inner metatarsal tubercle to tip of toe IV (FOL 13.1); distance from heel to tip of toe IV (TFOL18.1); relative length of toes, shorter to longest: I < II < III < V < IV (Figure 3E), tips of toes enlarged, rounded, webbing absent; subarticular tubercles rather indistinct; supernumerary tubercles weakly developed; inner metatarsal tubercle elongated (IMT 1.2). Skin of snout, between eyes, upper eyelids, side of head, and back have granular projections with horny spinules; dorsal part of limbs granular with horny spinules; throat shagreened to granular; chest, belly, and posterior surface of thighs granular.

*Color: in preservation*: dorsum light-brown, loreal and tympanic region light brown, lateral region dark grey with light colour stripes; fingers I and II, and toes I and II creamy white; ventral side light brownish grey with dark grey irregular spots; *in life*: dorsum golden yellow, lateral region brown with light yellow stripes extending from dorsum; fingers I and II, and toes I and II whitish; ventral side light grey with dark greyish brown irregular spots, lower jaw margins white.

#### Secondary sexual characteristics

Male (BNHS 5175) has nuptial spines on the prepollex; Ovary of single female specimen ((BNHS 4207) had 59 pigmented eggs, 1.2 ± 0.3 mm in diameter.

#### Variation

Measurements of eight type specimens are given in Table 1. During the breeding season, both male and female toads have a bright yellow dorsum with light yellow and light blue lateral stripes. Outside the breeding season, they have a light brownish grey dorsum with light yellow lateral stripes.

**Table 1:** Measurements of the two *Xanthophryne *gen. nov. species

*Xanthophryne tigerinus *sp. nov. (type series)												
	**Male**						**Female**					

	**BNHS** **5175**	**BNHS** **5177**	**BNHS** **4064**	**BNHS 4208**	**BNHS** **4209**	Mean	STDV	**BNHS** **5176**	**BNHS 4063**	**BNHS 4207**	Mean	STDV
SVL	30.5	31.0	32.9	32.0	27.8	30.8	1.9	33.9	33.3	35.3	34.2	1.0
HW	10.3	10.1	11.0	10.2	10.1	10.3	0.4	12.2	11.5	13.9	12.5	1.2
HL	10.1	10.0	10.7	10.1	9.0	10.0	0.6	11.2	10.9	12.2	11.4	0.7
IUE	2.3	2.1	2.3	2.1	2.5	2.3	0.2	2.3	2.5	3.0	2.6	0.4
UEW	3.1	2.9	3.6	3.1	2.9	3.1	0.3	3.4	3.4	3.2	3.3	0.1
SL	4.1	3.6	4.5	4.1	3.8	4.0	0.3	4.5	4.8	4.6	4.6	0.2
EL	3.1	3.0	4.0	4.0	3.0	3.4	0.5	4.4	4.6	4.5	4.5	0.1
FLL	6.1	6.8	5.4	6.5	5.8	6.1	0.6	7.4	6.1	6.1	6.5	0.8
HAL	7.0	6.9	6.7	6.8	6.7	6.8	0.1	6.7	6.9	7.4	7.0	0.4
ShL	11.8	11.9	11.8	11.7	10.4	11.5	0.6	11.6	11.7	12.2	11.8	0.3
TL	10.1	10.2	9.3	10.3	9.7	9.9	0.4	10.7	8.8	11.6	10.4	1.4
FOL	13.1	12.8	13.4	12.6	11.9	12.8	0.6	12.6	13.2	13.9	13.2	0.7

***Xanthophryne koynayensis***												

	**Males**											
	BNHS* 377	BNHS** 357	BNHS** 358	BNHS** 372	BNHS** 365	**BNHS** **5174**	**BNHS 5188**	Mean	STDV			
SVL	29.6	29.8	31.8	31.1	31.2	27.1	26.5	29.6	2.1			
HW	11.1	10.3	11.0	11.0	10.9	10.6	10.5	10.8	0.3			
HL	10.6	10.0	11.4	10.2	10.0	8.4	9.1	10.0	1.0			
IUE	2.4	2.0	2.4	2.4	2.2	2.5	2.7	2.4	0.2			
UEW	3.3	2.8	3.1	3.1	3.1	3.1	3.2	3.1	0.2			
SL	3.2	3.1	3.2	3.7	3.8	3.2	3.2	3.3	0.3			
EL	4.1	4.2	4.2	4.7	4.5	4.2	4.2	4.3	0.2			
FLL	6.2	5.3	5.8	5.8	6.2	5.2	6.0	5.8	0.4			
HAL	6.3	6.0	7.1	7.6	7.3	6.0	7.1	6.8	0.7			
ShL	11.8	10.1	11.5	11.3	11.5	10.2	11.2	11.1	0.7			
TL	11.9	10.0	11.6	11.2	11.6	10.1	11.3	11.1	0.8			
FOL	11.9	10.2	11.5	11.4	11.5	10.3	11.5	11.2	0.7			

* holotype and ** paratype of *Bufo sulphureus *Grandison and Daniel, 1964.

#### Distribution and natural history

**Figure 4:**
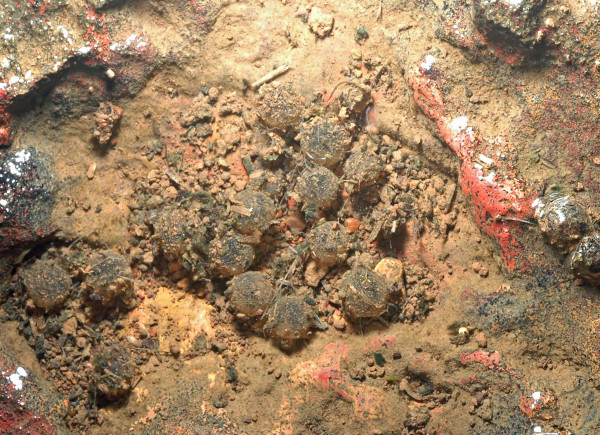
***Xanthophryne tigerinus *eggs are laid in clutches in temporary puddles**.

*Xanthophryne tigerinus *sp. nov. is known only from the type locality Amboli. The type series was collected from the ground near disturbed evergreen forest patches during a rainy evening after 19:00 h. Amplexus is axillary. Egg clutches contain 30-35 eggs per clutch (*N *= 4; VG, personal observation). The eggs are laid in temporary puddles on laterite rocks (Figure 4).

#### Notes

*Bufo koynayensis *Soman, 1963 was described twice based on the same type series but bearing different numbers. Soman first described *Bufo koynayensis*, without designating any type other than mentioning, "was collected in good numbers" [9]. Almost 40 years later, specimen ZSI A.1784 was recognized as the holotype of *Bufo koynayensis *Soman, 1963 [10]. This specimen was examined and is badly damaged (SDB, personal observation). The samples collected by Soman were utilized to describe *Bufo sulphureus *(holotype, BNHS 377 and 26 paratypes including 5 males, 11 females and 10 juveniles) [11], which as a consequence is a junior synonym of *Bufo koynayensis*. As described in the original description, the type series of *Bufo sulphureus *were deposited in the British Museum of Natural History, the Indian Museum and the Bombay Natural History Society. In this study, we examined the holotype and 18 paratypes (14 adult males - SVL 24.0-31.8 mm and 4 subadults - SVL 20.9-25.0 mm) deposited in BNHS and five paratypes in BMNH under the nomen 'Bufo *sulphureus'*. In addition, recent collections of 'Bufo' koynayensis from the type locality were compared. All available material of this species is consistent with the original description.

## Conclusion

*Ghatophryne *gen. nov. and *Xanthophryne *gen. nov. are both endemic to the Western Ghats of India. Together with the arboreal toad genus *Pedostibes *(Western Ghats endemic; species from South-East Asia belong to another clade) and the genus *Adenomus *(Sri Lanka endemic), they form the oldest lineages in the Adenominae clade. The recognition of each of these lineages as distinct genera highlights their early diversification on the southern parts of the Indian subcontinent [5].

## Competing interests

The authors declare that they have no competing interests.

## Authors' contributions

All authors contributed equally, and read and approved the final manuscript.
